# Species Recognition and Cryptic Species in the *Tuber indicum* Complex

**DOI:** 10.1371/journal.pone.0014625

**Published:** 2011-01-28

**Authors:** Juan Chen, Shun-Xing Guo, Pei-Gui Liu

**Affiliations:** 1 Key Laboratory of Biodiversity and Biogeography, Kunming Institute of Botany, Chinese Academy of Sciences, Kunming, Yunnan, People's Republic of China; 2 Institute of Medicinal Plant Development, Chinese Academy of Medical Sciences and Peking Union Medical College, Beijing, People's Republic of China; University of Wisconsin-Milwaukee, United States of America

## Abstract

Morphological delimitation of Asian black truffles, including *Tuber himalayense*, *T. indicum*, *T. sinense*, *T. pseudohimalayense*, *T. formosanum* and *T. pseudoexcavatum*, has remained problematic and even phylogenetic analyses have been controversial. In this study, we combined five years of field investigation in China with morphological study and DNA sequences analyses (ITS, LSU and β-tubulin) of 131 *Tuber* specimens to show that *T. pseudohimalayense* and *T. pseudoexcavatum* are the same species. *T. formosanum* is a separate species based on its host plants and geographic distribution, combined with minor morphological difference from *T. indicum*. *T. sinense* should be treated as a synonym of *T. indicum*. Our results demonstrate that the present *T. indicum*, a single described morphological species, should include at least two separate phylogenetic species. These findings are of high importance for truffle taxonomy and reveal and preserve the richness of truffle diversity.

## Introduction

*Tuber* F. H. Wigg., an ectomycorrhizal genus in the *Pezizales* (*Ascomycota*), is presently represented by 16 species in China, 8 of which are endemic to Yunnan and Sichuan [1]. This area belongs to an important part of the Hengduan Mountains, regarded as one of the world's 34 biodiversity hotspots [2]. Several new species (*T. sinense, T. formosanum, T. pseudohimalayense* and *T. pseudoexcavatum*), morphologically similar to *T. indicum* (Asian black truffle) and *T. melanosporum* (European Périgord black truffle), have been reported in China [3], [4], [5], [6]. These species were commonly characterized by dark-brown to black ascocarps with conspicuous peridial warts and spiny or spinose-reticulate ascospores.

Several scholars have explored the phylogenetic relationships between Asian and European black truffles and clarified the taxonomy of *T. indicum* and *T. melanosporum*. However, remarkable similarity in appearance among the members of Asian black truffles (including *T. indicum, T. himalayense, T. sinense, T. formosanum* and *T. pseudohimalayense*) make it difficult to discriminate between species. Most previous studies have shown that two groups exist in Asian black truffles (the groups A and B), but taxonomic treatment of the two groups has still remained controversial. Some scholars have proposed that the two groups (A and B) belonged to two distinct species, *T. himalayense* and *T. indicum*, respectively [7], [8], [9], while others have suggested that they are two geographical ecotypes of one species [10], [11]. These discrepancies might originate from the high morphological and molecular variability within the *T. indicum* complex and the insufficient number of samples studied.

Ascosporic characters, especially ornamentation on the surface of the mature ascospores, are an important diagnostic trait for the genus *Tuber* in classical taxonomy [12], [13], [14]. For example, species of *Tuber* can be divided easily based on spore ornamentation into three groups: spore reticulate, spore spiny and spore spinose-reticulate [1]. Discrimination between *T. indicum, T. himalayense* and *T. pseudohimalayense* in the previously reported studies depended mainly on spore ornamentation [5], [15]. However, wide ornamentation variability is displayed in inter- or intraspecies. Moreover, a larger number of fruit bodies marketed as *T. indicum* have been rarely examined microscopically.

Therefore, it is necessary to carry out a thorough taxonomical study on these Asian black truffles species. By analyzing the type or isotype specimens and a larger number of Chinese materials with known exact origin, we aimed to accomplish the following : (i) assess the reliability of spore ornamentation as one of important diagnostic characters in distinguishing the black truffles; (ii) phylogenetically analyze ribosomal DNA sequences (LSU, ITS) and β-tubulin to clarify whether these taxa are conspecific; and (iii) define species taxonomic boundaries and summarize the intraspecific variability of these taxa based on morphological, molecular, geographical and ecological traits.

## Results

### Morphological analyses

**Figure 1:**
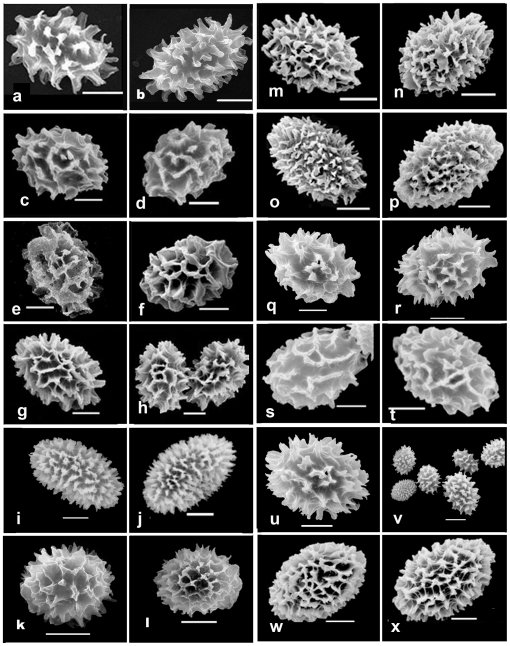
SEM of *Tuber* species ascospores, showing the detail of ornamentation. a–b. *T. indicum* **Holotype** K(M)39493; c–d. *T. sinense* **Isotype** HMAS60222; e–f. *T. himalayense* **Isotype** K(M)32236; g–h. *T. formosanum* KUN-HKAS49707; i–j. *T. melanosporum* KUN-HKAS52034; k–l. *T. pseudohimalayense* **Isotype** AH18331; m–n. *T. indicum* KUN-HKAS29357; o–p. *T. indicum* KUN-HKAS30261; q–r. *T. indicum* KUN-HKAS52030; s–t. *T. indicum* KUN-HKAS44330; u–v. *T. indicum* KUM-HKAS44988; w–x. *T. indicum* KUN-HKAS41312.

**Figure 2:**
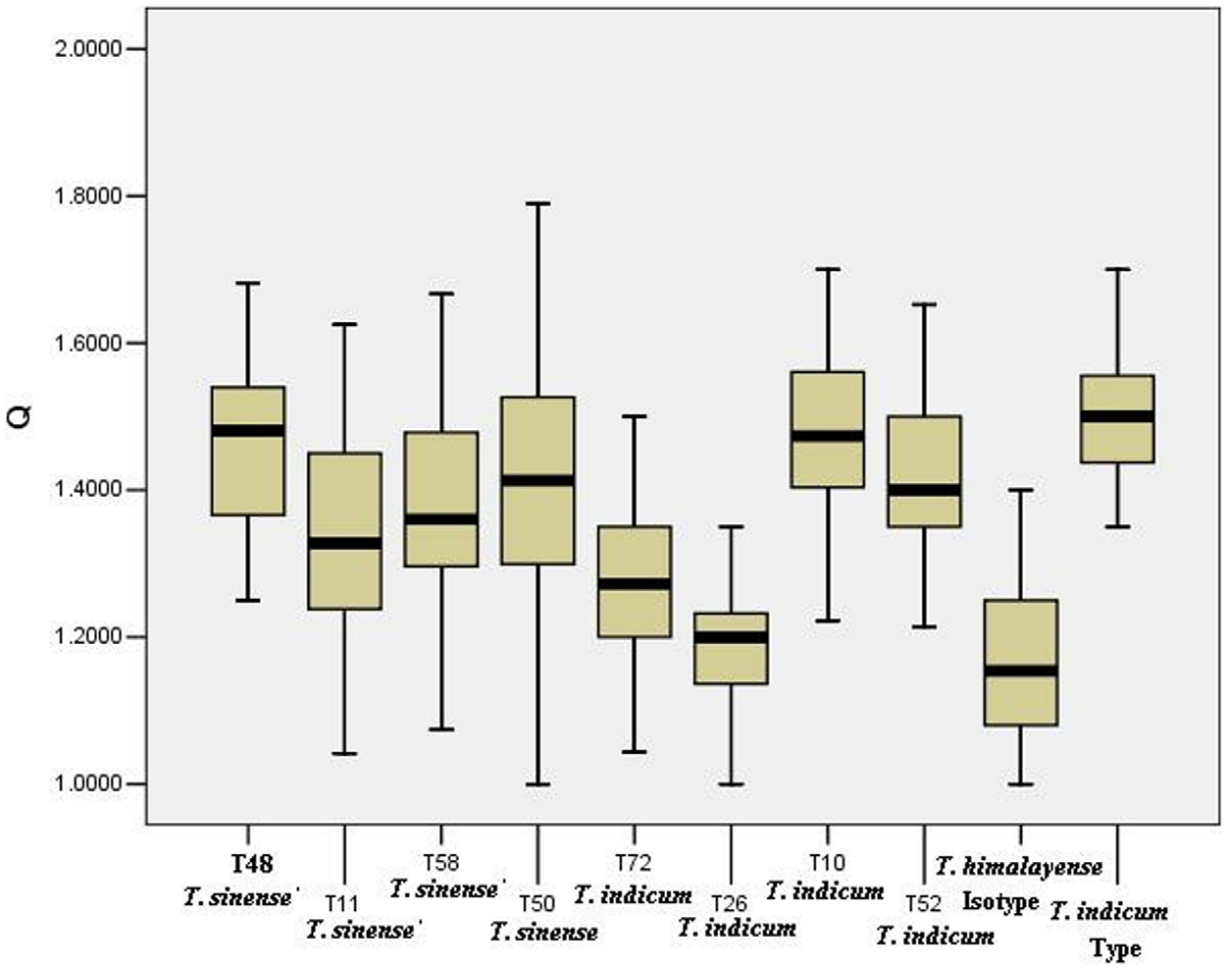
Box plot of length/width ratio (Q) of an ascospore. Box plot illustrate the medians, upper and lower quartiles, range and extreme values. 5–7 fruit bodies and 300 measurements of each typical specimens of *T. indicum*. One fruit body of *T. indicum* holotype (100 measurements) and half fruit body of *T. himalayense* isotype (100 measurements).

Ascocarp surface characters (color and warts), peridium structures and spore ornamentations were examined in detail. First, morphological analyses based on *T. indicum* type and isotypes of *T. himalayense*, *T. sinense* and *T. pseudoexcavatum* specimens showed that *Tuber pseudohimalayense* and *T. pseudoexcavatum* have nearly identical peridia, asci and ascospores. For example, the peridium of the *T. pseudohimalayense* isotype was covered with minute, pyramidal warts (0.5–1.5 mm wide). The outer peridial layer was pseudoparenchyma and composed of subglobose to ellipsoid cells 10–30 µm diam. The asci usually contained 1–7(8) spores ornamented with spines connected by low ridges to form an alveolate reticulum. The reticulum was usually regular and less than 1 µm tall, and the spine was 5–7 µm tall (Figure 1k, l). These characteristics differed conspicuously from those of *T. indicum, T. sinense, T. formosanum* and *T. himalayense* (Figure 1). Second, *T. himalayense* was characterized by broadly ellipsoid spore (Q<1.3) ornamented frequently with irregular reticulum ornamentation (3–5 µm height) and seldomly spines; these morphological traits distinguished it from *T. indicum* and *T. formosanum* [ellipsoid spore (Q≥1.3) ornamented with free spines] (Figure 1 and Figure 2). Third, *T. formosanum*, originally described from Taiwan, was characterized by black ascocarp with apex warts and spores with free straight spine or irregular reticulum, which discriminated it them from other species (Figure 1g, h). Finally, we could not discriminate *T. indicum* from *T. sinense* based on our morphological examination because many morphological traits displayed a continuum or overlap when a large number of Chinese specimens were observed and analyzed. For example, in most cases, some specimens have spores ornamented with sparsely or densely free spine (5–10 across the spore width), and some cases have spores with incomplete reticulum or ridge formed by spine weakly connected each other at the base (such as *T. sinense*) (Figure 1m–x). Moreover, the shape and size of spores between *T. indicum* and *T. sinense* also displayed a continuum and overlapped (Figure 2).

### Phylogenetic analyses

In the present study, most new sequences were derived from fresh samples collected by our lab. The holotype of *T. indicum* [K (M) 39493] and the isotype of *T. himalayense* [K(M)32236] produced no sequences due to limitation of the long storage period and the small amount of sample. Only the LSU sequence for *T. pseudohimalayense* isotype was available to us because PCR amplification for ITS and β-tubulin failed. Genetic distances were calculated from ITS, LSU and β-tubulin sequences. For the ITS data, the average similarity within each group of the *T. indicum* complex was 99.35% (SD = 0.02), whereas that among the groups of the *T. indicum* complex was 96.41% (SD = 2.15). For the β-tubulin data, the average similarity within each group of the *T. indicum* complex was 99.84% (SD = 0.157), whereas that among the groups of the *T. indicum* complex was 99.26% (SD = 0.283).

**Figure 3:**
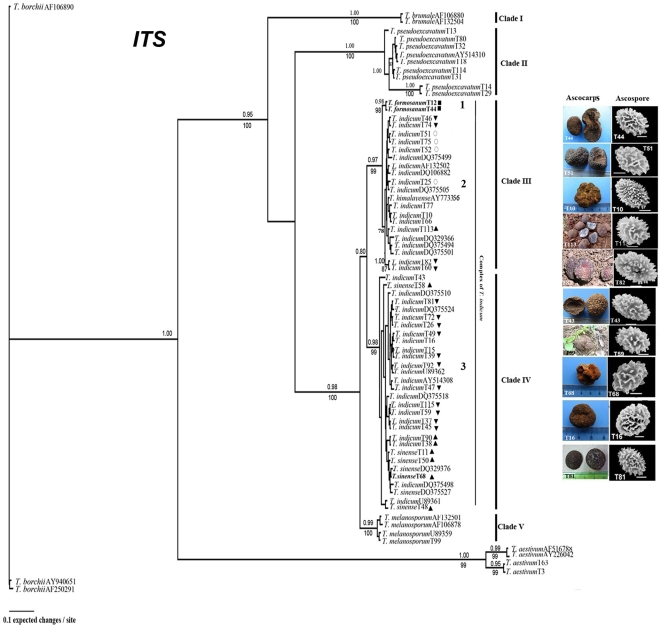
Bayesian 95% majority-rule tree for Asian black truffles as inferred from ITS sequences. Numbers above branches indicate posterior probabilities (>95%) and numbers below branches are bootstrap values (≥70%) from 1000 replicates. Bold represents isotype specimens or specimens from type locality. Symbols beside OUT names indicate host plant species as follows: closed triangles pointing up, *Pinus armandii*; closed triangles pointing down, *Pinus yunnanensis*; closed squares, *Cyclobalanopsis glauca*; open ellipsoid, *Castanea mollissima*. OUT refers to the population names indicated in Table S1.

The ITS sequence alignment contained 74 sequences (including three outgroups) and 950 characters, of which 436 were parsimony-informative. The MP and Bayesian analyses conducted with similar topology; thus, only the Bayesian tree is shown in Figure 3. All *Tuber* samples analyzed fall into five major clades (Figure 3). *Tuber* complex (*T. indicum,T. formosanum* and *T. sinense*) belong to two large clades (Clade III and IV) with high posterior probabilities and bootstrap values (PP>95% and BP = 99%). Clade III includes *T. formosanum* and *T. indicum*. *T. formosanum* formed a well-supported subclade within Clade III with both 98% PP and PP values (group 1), and *T. indicum* formed another subclade with weak support (78% BP, group 2). Clade IV includes *T. sinense* and *T. indicum*. *T. pseudoexcavatum* formed a significant supported monophyletic group (Clade II) with 100% PP and 100% BP values. *T. melanosporum*, a European black truffle with ascocarps of conspicuous warts and densely spiny ascospores (spine 2–3 µm high), formed a monophyletic clade with 99% support and grouped closely to the *T. indicum* complex (Clade V).

**Figure 4:**
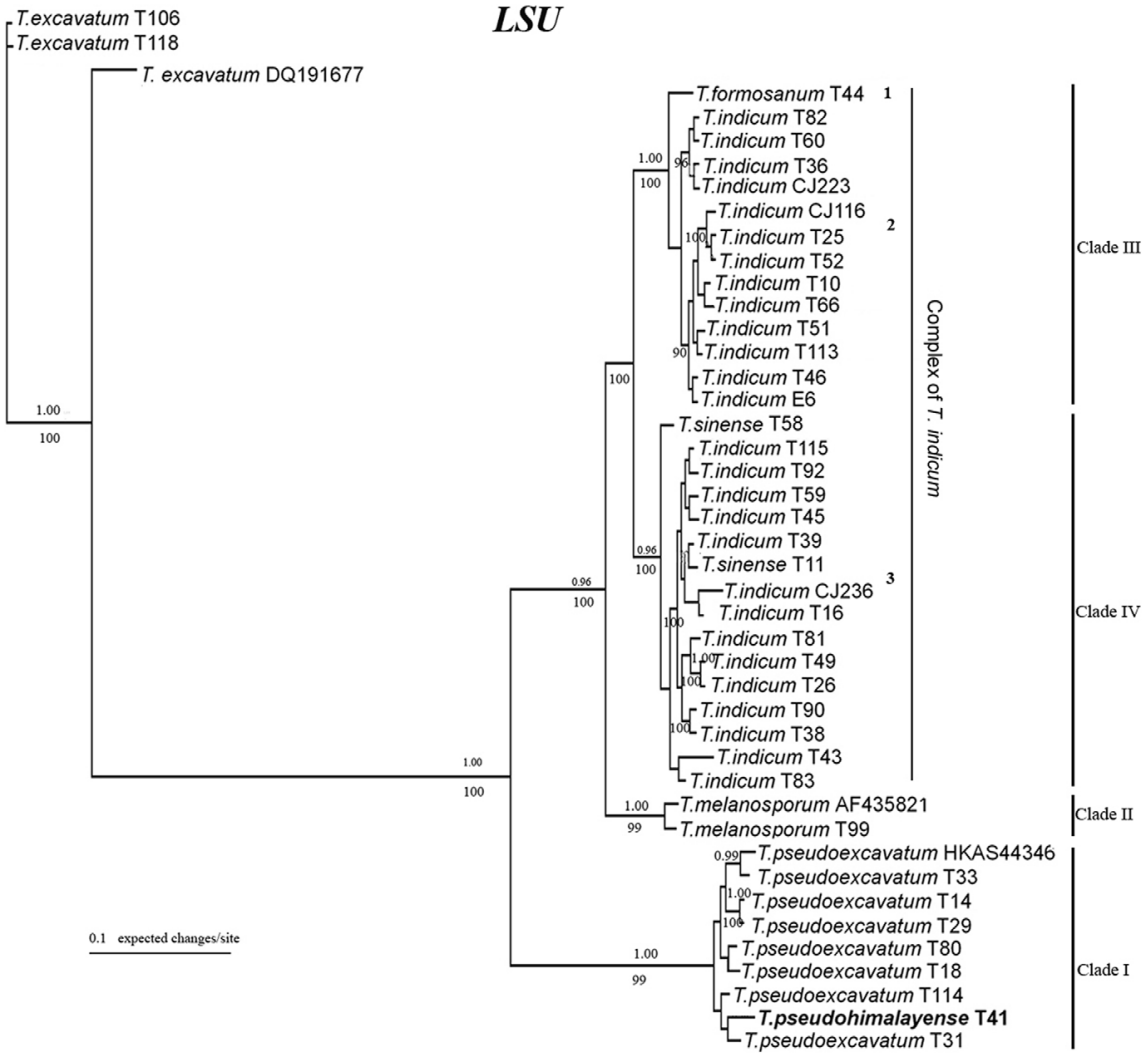
Phylogenetic relationship for Asian black truffles inferred from LSU by Bayesian analyses. Numbers above branches indicate posterior probabilities (>95%) and numbers below branches are bootstrap values (≥70%) from 1000 replicates. Bold represents isotype specimens.

The LSU data set comprised 44 taxa, 850 total characters and 119 parsimony-informative characters. The Bayesian analysis tree is shown in Figure 4. MP analyses (28 trees, TL = 148, CI = 0.838, RI = 0.960) revealed topologies very similar to those obtained by Bayesian analyses. The existence of two large clades of the *T. indicum* complex (Clade III and Clade IV) also were both supported by 100% BP and PP. Similar to the phylogram generated from the ITS tree, specimens morphologically identified as “*T. indicum*” were divided into two groups (group 2 and 3). *T. sinense* was nested within Clade IV (group 3) with part of the *T. indicum* samples. *T. pseudohimalayense* and *T. pseudoexcavatum* were clustered and formed a well-supported monophyletic group with 100% PP support.

**Figure 5:**
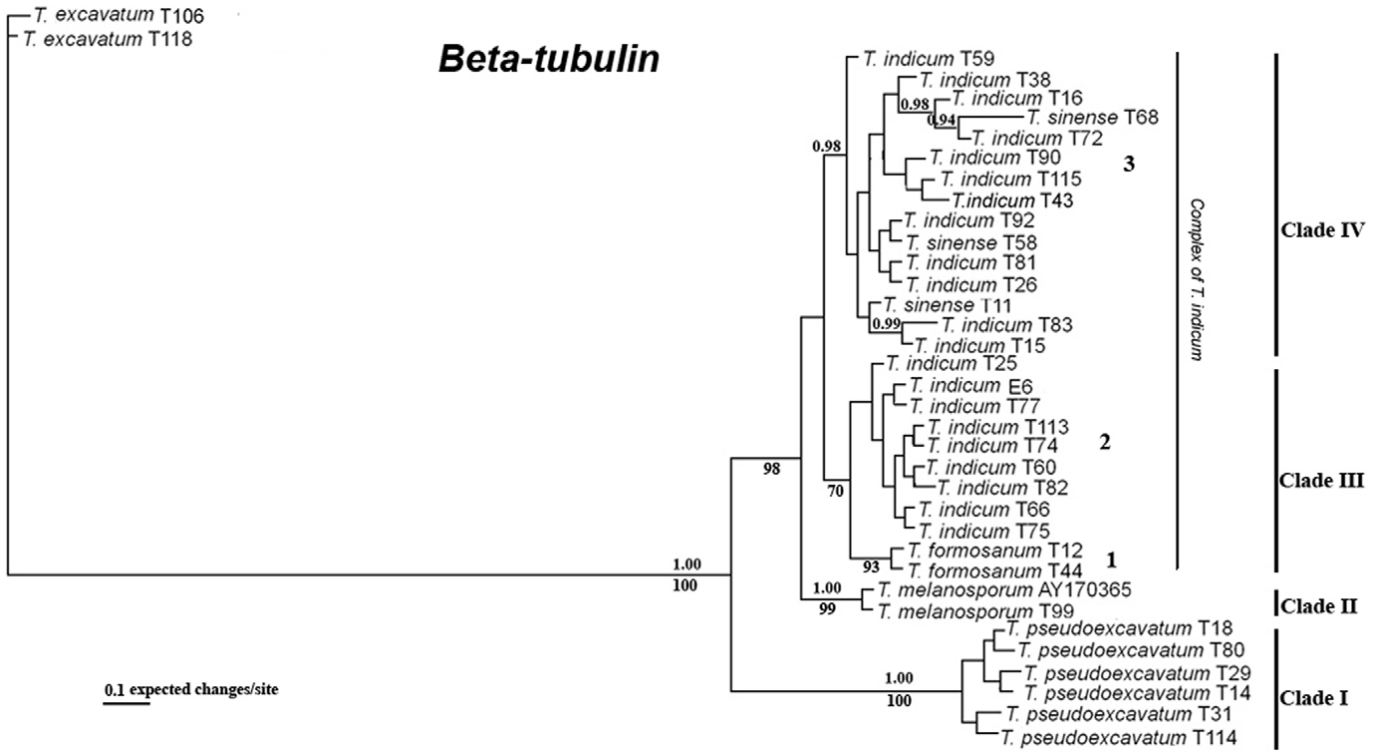
Phylogenetic relationship for Asian black truffles inferred from β-tubulin by Bayesian analyses. Numbers above branches indicate posterior probabilities (>95%) and numbers below branches are bootstrap values (≥70%) from 1000 replicates.

The β-tubulin data consisted of 36 taxa and 615 characters and 14 ambiguous characters were excluded from the analyses. Of the remaining 601 characters, 508 characters were constant, and 81 were parsimony-informative characters. The Bayesian inference topology is shown in Figure 5. The MP trees (78 tree, TL = 73, CI = 0.959, RI = 0.976) had nearly identical topologies. Similar to the ITS and LSU topology, the Bayesian analyses conducted using the β-tubulin sequence divided all samples into four well-supported clades: two large clades (three groups) of the *T. indicum* complex, the *T. melanosporum* clade and the *T. pseudohimalayense*/*T. pseudoexcavatum* clade. Specimens morphological identified as “*T. indicum*” formed two groups, of which group 3 had 98% PP support and group 2 had below 90% PP support (data not shown).

**Figure 6:**
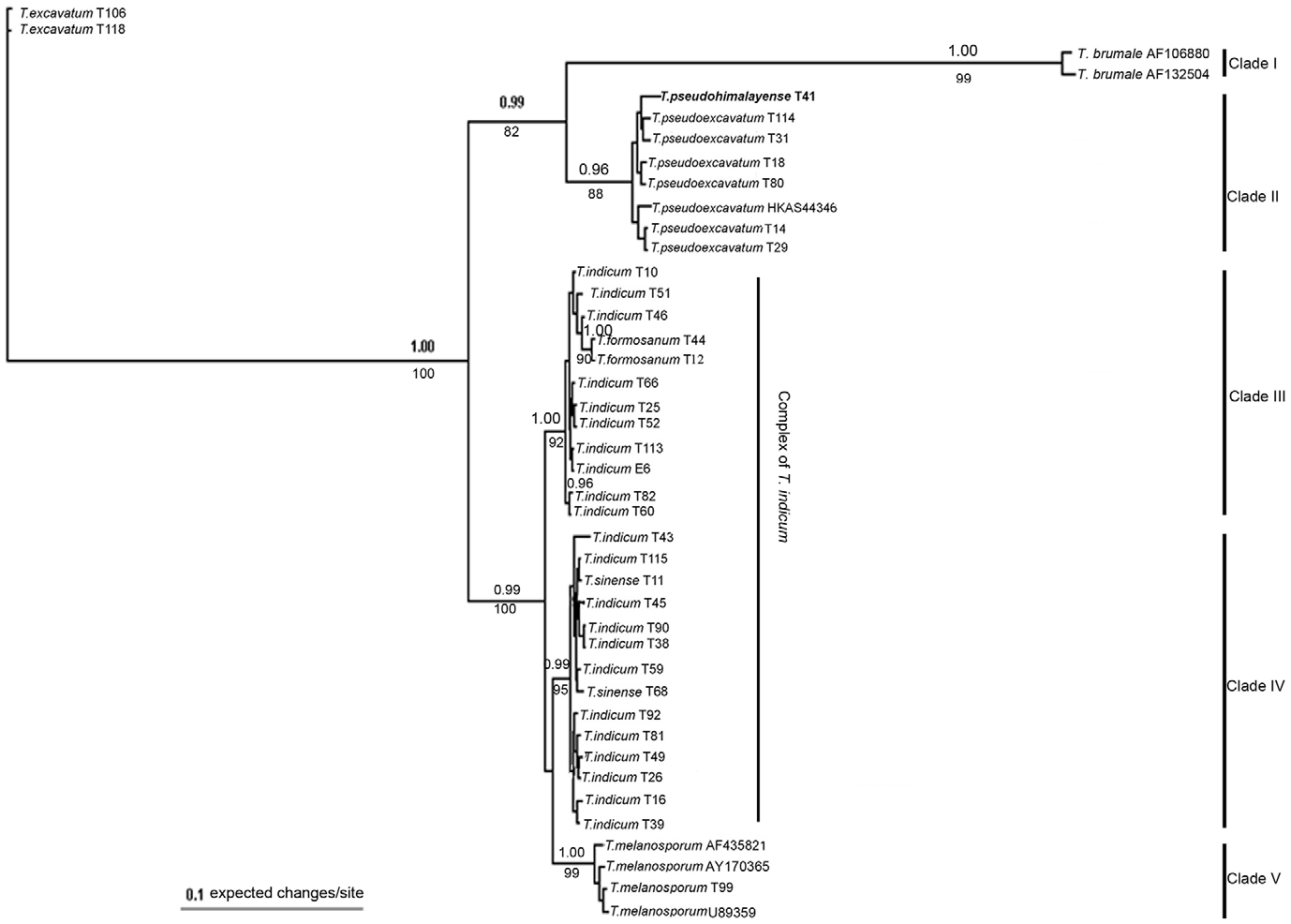
Bayesian 95% majority-rule tree from combined nucleotide sequences. Numbers above branches are posterior probabilities (>95%) and numbers below branches are bootstrap values (≥70%) from 1000 replicates.

The combined data set of three loci comprised 42 sequences because some specimens were sequenced successfully on only one of three loci. We selected those samples that had at least two loci sequences to perform the combined analyses with an exception of *T. pseudohimalayense*. The combined analyses include 1872 characters, of which 305 were parsimony-informative. Bayesian and maximum parsimony analyses revealed identical topologies. Both analyses strongly support the existence of two large monophyletic clades within the *T. indicum* complex (Figure 6). However, for *T. formosanum*, and *T. melanosporum*, the topologies of the combined loci conflicted with the phylogram of the individual loci. On the ITS, LSU and β-tubulin sequences, *T. formosanum* formed a single subclade within clade III, and *T. melanosporum* formed monophyletic Clade VI or III and grouped sister to the *T. indicum* complex. On the combined loci, *T. melanosporum* and some samples of *T. indicum* were clustered together and formed a group.

Specimens morphologically identified as “*T. indicum*” were divided into two groups (group 2 and 3) based on individual ITS, LSU and β-tubulin sequences and combined loci. Moreover, no conspicuously morphological differences were detected within the two groups. For example, some samples with ornamented spores and that were typically spine-free (e.g., T10 and T81) fell into both group 2 and 3 (Figure 3). Similarly, some samples with irregular or incomplete spinose-reticulate spore (e.g., T51, T113, T16 and T68,) also were included in the two groups (Figure 3). Furthermore, samples of both *T. indicum* groups showed great variations in spore shape [from broadly ellipsoid (Q<1.3) to ellipsoid (Q≥1.3) (Figure 2).

### Host plant differences among the *T. indicum* complex lineages

In the present study, we identified host plants using morphological observations. Our collection sites were typically pure forests composed of species in Pinaceae (*Pinus yunnanenses* or *Pinus armandii*). Although a few collection sites were mixed forest composed of species in Fagaceae (*Quercus acutissima)* and Pinaceae, *Tuber indicum* is probably associated with *Pinus* host plant based on the morphological characteristics of the mycorrhizae. In addition to Pinaceae hosts, Fagaceae hosts (*Castanea mollissima*) were found to associate with *T. indicum*. Furthermore, some samples of *T. indicum* were collected from a *Castanea mollissima* plantation.

**Figure 7:**
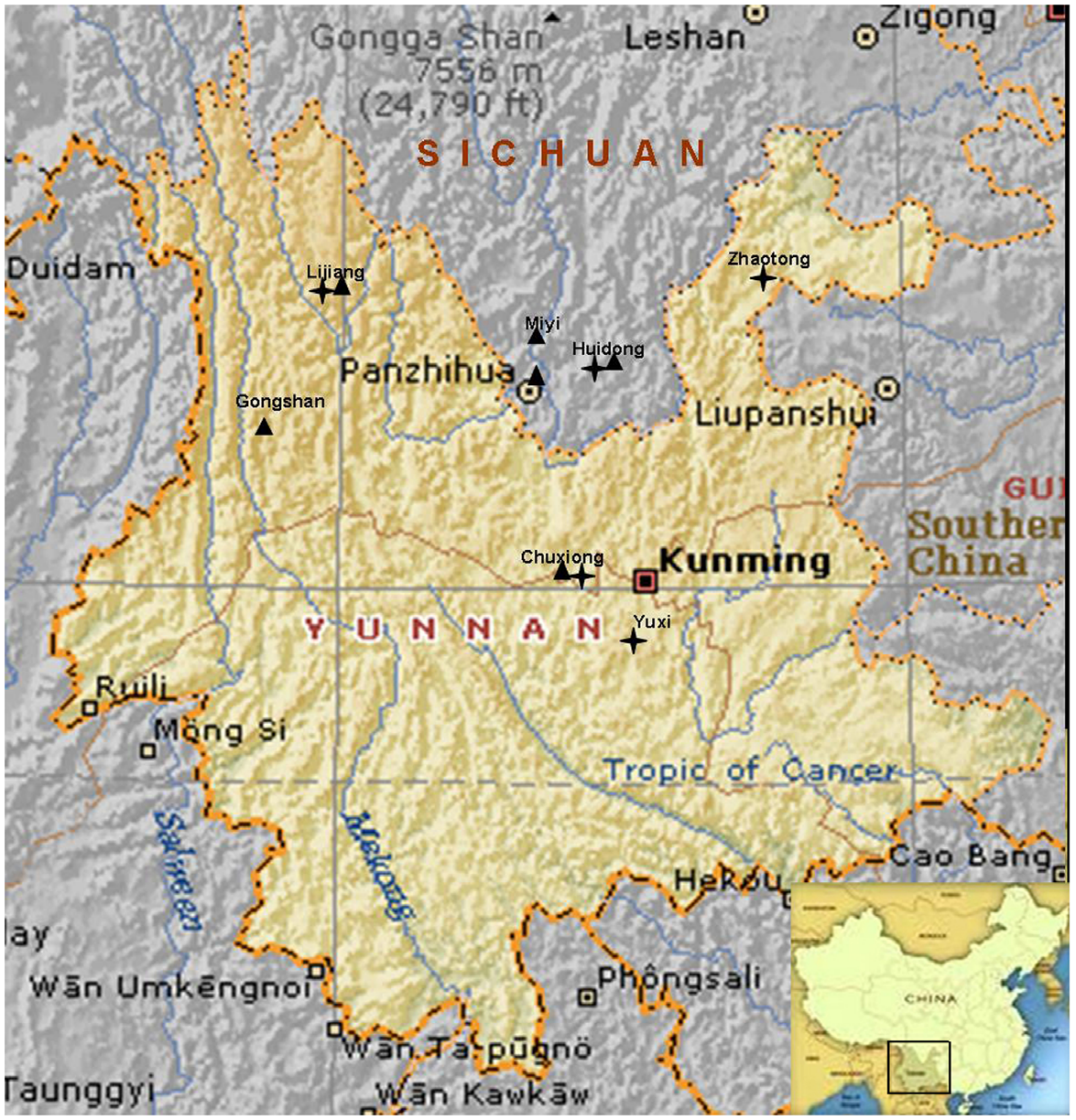
Sampling localities of the two groups within specimens morphological identified as *T. indicum* in **Figure 3**. Group 2 in Clade III (black triangles) and group 3 (Clade IV) (black star).

Neither a host specialty nor a geological specialty was found between the two large clades of the *T. indicum* complex (Clade III and Clade IV in Figure 3 and 7). One lineages of the *T. indicum* complex (Clade III in Figure 3), including *T. indicum* and *T. formosanum*, had both had Fagaceae and Pinaceae as host plants. *T. formosanum* associated with *Cyclobalanopsis glauca*, whereas *T. indicum* associated with *Castanea mollissima* and *Pinus armandii* and *P. yunnanenses*. Another lineage of the *T. indicum* complex (Clade IV in Figure 3), including *T. indicum* and *T. sinense*, associated only with *Pinus armandii* and *P. yunnanenses*. Moreover, with the exception of *T. formosanum* (distributed in Taiwan Island), samples in the two groups of “*T. indicum*” have overlapped distribution (Figure 7).

## Discussion

The broad variability in morphological characteristics as well as the difficulties of cultivating and the impossibility of mating these symbiotic *Tuber* specimens under controlled conditions has made it difficult to determine the interspecific or intraspecific boundaries [16]. Thus, it is necessary to combine morphological and molecular traits when grouping truffles with indistinguishable or divergent morphological traits into species, subspecies, or varieties.

Species of Asian black truffles, including *T. indicum, T. sinense, T. himalayense, T. formosanum* and *T. pseudohimalayense*, have been confused due to the lack of sufficient distinguishing morphological characters. The phylogenetic analyses conducted in previous publications using the genetic markers ITS-RFLP, ITS regions and β-tubulin have suggested that two clades exist in Asian black truffles [7], [ 8], [ 9], [ 10], [ and 11]. Regarding these results, some mycologists have provided two different views: that two separate species exist (*T. indicum* and *T. himalayense*) or that a single species exists (*T. indicum*) based on a morphological concept. *T. pseudohimalayense* has been treated as a synonym of *T. indicum* in previous studies [9], [11]. Recently, Manjón et al. proposed that *T. pseudohimalayense* is a synonym of *T. pseudoexcavatum* based on its mtLSU sequence [17].

Morphological and molecular analyses based on LSU and the *T. pseudohimalayense* isotype in the present study showed that *T. pseudohimalayense* and *T. pseudoexcavatum* are exactly the same species, similar to the results presented by Manjón et al. Although the name *T. pseudoexcavatum* is much more widely used in the recent truffle literature than *T. pseudohimalayense*, the latter has priority over *T. pseudoexcavatum*. Our results clarify the taxonomic confusion between *T. pseudohimalayense* and *T. indicum* and provide a necessary complement to previous studies.

The multigene phylogeny based on ITS, LSU and β-tubulin indicates that the *T. indicum* complex (*T. indicum*, *T. sinense* and *T. formosanum*) are not a monophyletic group and include at least two large monophyletic clades. The two clades are morphologically indistinguishable from each other, and no significant differences between on host plants and geological distribution were detected between them with exception of *T. formosanum*. *T. indicum* and *T. sinense* have been confused due to their great morphological similarity. *T. sinense* differs from *T. indicum* in that *T. sinense* has an incomplete reticulum on the ascospores surfaces formed by spines that widen at their base, which is included in its original description [3]. In fact, our morphological observations based on isotype specimens showed that *T. sinense* could represent a morphologically intermediate type of *T. indicum,* and molecular data suggest that *T. indicum* and *T. sinense* should be conspecific.

A previous study based on phylogenetic methods, which are probably the most common and useful in species delineation, indicate that there might be many cryptic species or phylogenetic species in higher fungi [18]. The present study used a simple approach, consisting of fulfilling one of two criteria, to identify independent evolutionary lineages and phylogenetic species from multilocus genealogies. Using the first criterion, genealogical concordance, the clade was present in the majority of the single-locus genealogies. Using the second criterion, genealogical non-discordance, the clade was well supported in at least one single-locus genealogy, as judged by both MP bootstrap proportions above 70% and Bayesian posterior probabilities above 95% and was not contradicted in any other single-locus genealogy at the same level of support [19], [20]. Our results based on ITS, LSU and β-tubulin sequence analyses showed that specimens morphological identified as “*T. indicum*” apparently include at least two cryptic species (group 2 and group 3) and that the two groups of *T. indicum* have broad morphological variations, although definition of group 2 lacks power (PP and BP value<50%). A possible explanation for this result might be the small sample size.

The failure to obtain DNA sequences from the *T. indicum* type and the *T. himalayense* isotype made it difficult to interpret the phylogenetic relationship between *T. himalayense* and other Asian black truffles. However, unique morphological traits of the *T. himalayense* isotype (broadly ellipsoid spore ornamented irregular spinose-reticulum) could help distinguish them from *T. indicum* (ellipsoid spore with sparsely spine). Until now, none of additional collections, which morphologically resemble to isotype of *T. himalayense*, were reported all over the world since the first publication. We reexamined a Chinese specimens, which has been appointed to be *T. himalayense* (HKAS 25689, GenBank accession number AY773356) in a previous study [9] and found that the specimen was immature. Furthermore, the few immature spores were ornamented mostly with spines rather than with irregular reticulum, which is found in *T. himalayense*. Moreover, those specimens (e.g., T60, T82 and T10) that grouped with *T. himalayense* (AY773356) within the same clade in the phylogenetic tree (Figure 3) were morphologically similar to *T. indicum*. Therefore, it might be unreasonable to treat one of two clades of the *T. indicum* complex as *T. himalayense* as has been done in previous studies [9]. The exactly phylogenetic relationship between *T. himalayense* and other related species needs further investigation. In the present study, we recognized the species based on its morphological traits.

In conclusion, we addressed the taxonomic relationship between Asian black truffles (*T. indicum, T. himalayense*, *T. sinense*, *T. formosanum* and *T. pseudohimalayense*) using morphological and phylogenetic methods. *T. pseudohimalayense* and *T. pseudoexcavatum* were confirmed to be the same species. *T. formosanum* had a few morphological difference (black ascocarp with apex warts and spores with free straight spine) and a special host plant (*Cyclobalanopsis glauca*) and distribution (Taiwan island) that differed from *T. indicum* and, thus, was treated as a separate species. *T. sinense* was treated as a synonym for *T. indicum* and *T. indicum* included at least two phylogenetic or cryptic species. Our morphological analyses illustrated that the ascomata surface configuration (e.g., color, shape and wart), peridium and the shape and ornamentation of the ascospore were still important diagnostic factors in Asian black truffles although these features were inconspicuous for identifying *T. indicum* and *T. sinense*.

## Materials and Methods

### Fungal samples

Over the past five years, we extensively investigated distribution of *Tuber* spp. in China, especially southwestern region. Ninety-four *T. indicum/T. sinense* specimens (see Text S1) and 39 *T. pseudoexcavatum* specimens from China, the holotype of *T. indicum* [K(M)39493], the isotypes of *T. himalayense* [K(M)32236] (originally described from India), *T. sinense* (HMAS60222), *T. pseudohimalayense* (AH18331) (originally described from China) and 2 specimens of *T. formosanum* from Taiwan type locality(HKAS49707) were carefully morphologically reexamined. Additionally, 12 specimens from Europe, including 6 species (*T. melanosporum, T. brumale, T. aestivum, T. borchii, T. excavatum* and *T. rufum*) and 2 species (*T. spinoreticulatum* and *T. lyonii*) from North America also were compared morphologically.

### Morphological observation

Ascocarps surface configuration (color and warts), peridium structure and spore shape, size and ornamentation at maturity were observed and recorded in detail [21]. Morphologically identification as *T. indicum* or *T. sinense* was performed under a microscope according to characters of *T. indicum* holotype and *T. sinense* isotype. Those specimens collected from type of locality typical for *T. sinense* were labeled as “*T. sinense*”. Microscopic features (especially ascospores) were examined using a light microscope (Nikon E400) and a scanning electron microscope (SEM). For SEM, spores were scraped from the dried gleba and mounted in distilled water on a cover glass; when dry, the cover glass was pasted directly onto an SEM stub with double-sided tape, coated with gold-palladium and photographed with an AMRAY 1000B SEM [22]. Herbaria are abbreviated according to Index Herbariorum [23].

### DNA extraction, PCR amplification and nucleotide sequencing

Based on morphological examination, at least 55 *Tuber* samples were selected for molecular analyses (excluding sequences from Genbank database). Samples were chosen to represent the morphological, ecological and geographical ranges of *T. indicum.* In addition, *T. aestivum* collected from Sichuan Province of China specimens and usually marketed as *T. indicum* collected from the local market and *T. melanosporum* from Italy were studied and sequenced (see Table S1). Genomic DNA was extracted from dried or fresh material with the E.Z.N.A. Fungal DNA kit (Omega Bio-Tek, Doraville, Georgia) according to the manufacturer's protocol.

Internal transcribed spacer regions (ITS), the large subunit ribosomal rRNA (LSU) and β-tubulin were amplified with primers pairs ITS4 and ITS5 [24], primers pairs LR5 and LROR [25] and primers pairs Bt2a and Btspect [7], [26], respectively. PCR products were purified using Watson's PCR purification kit (Watson, China). Sequencing was performed with a BigDye Terminator sequencing kit (Applied Biosystems, USA) and analyzed with an ABI 3730 automated sequencer (Applied Biosystems, USA). Sequence chromatograms were compiled with Sequencher 4.1 software.

### Sequence alignments and phylogenetic analyses

Sequences were edited with SeqMan (Dnastar Package). Nucleotide sequences were initially aligned with Clustal X 1.83 [27] and adjusted manually in BioEdit Version 5.0.9 [28]. Maximum parsimony(MP) analyses were conducted using PAUP version 4.0b10 [29] on G5 Macintosh computers by heuristic search and tree bisection reconnection (TBR) branch swapping with 1000 search replicates, each replicate with random sequence addition. In all analyses, gaps were treated as missing data, character states were treated as equally weighted and unordered. A total of 1000 bootstrap replicates were performed for each maximum parsimony analyses.

Bayesian analyses were conducted with MrBayes 3.1.2 [30]. The best-fitted evolutionary model was estimated using MrModeltest v. 1.01 [31]. *T. borchii* and *T. excavatum* were used as outgroups. An initial run for 2,000,000 generations with four simultaneous Markov chain Monte Caro (MCMC) chains was performed to estimate how many generations were required for likelihood scores to reach stationary. At the end of each run, we considered the sampling of posterior distribution to be adequate if the average standard deviation of split frequencies was less than 0.01. Bayesian PP was determined by computing majority rule consensus trees by use of the set of trees that reached stationary phase. A probability of 95% was considered significant.

## Supporting Information

Text S1 Ninty-four Voucher specimens of T.indicum/T.sinense examined in the study.
(0.03 MB DOC)

Table S1 Tuber species used in the study and their geographic distribution.
(0.22 MB DOC)
