# Supplementary material for: Species Recognition and Cryptic Species in the Tuber indicum Complex
Source: PLoS One. 2011 Jan 28;6(1):e14625. doi: 10.1371/journal.pone.0014625 (PMC3030557; doi:10.1371/journal.pone.0014625)
Supplement: Text S1 — Ninty-four Voucher specimens of T.indicum/T.sinense examined in the study. (0.03 MB DOC) [file pone.0014625.s001.doc]

**Text S1. Ninty-four Voucher specimens of *T.indicum/T.sinense* examined in the study.**

*Specimens examined:* China**.** Sichuan Prov.: Panzhihua city, 16 May 1995, *C. J. Gu 1* (KUN-HKAS29366); *C. J. Gu 2* (KUN-HKAS29359); 16 Oct 1998, *M.Yang 1* (KUN-HKAS32849); 10 Nov 1996, *D. S. Wang 2* (KUN-HKAS30261); *D. S. Wang 3* (KUN-HKAS30262); Jan 2000, *M. Q. Gong2* (KUN-HKAS35995); *M. Q. Gong 3* (KUN-HKAS35996). Huidong County, Xiaochahe, 13 Sept 1988, *K. Tao & D. C. Zhang* (MHSU1633 – **Isotype** of *T. sinense*); Huidong County, Jiangzhou, 2100m, 22 Oct 2003, *CJ138* (KUN-HKAS44327); Huidong County, Gaji, 1820m, 22 Oct 2003, *CJ143* (KUN-HKAS44342); *CJ139* (KUN-HKAS44319); *CJ132* (KUN-HKAS44345); *CJ155* (KUN-HKAS44992); *CJ136* (KUN-HKAS44328); *CJ133* (KUN-HKAS44326); Huidong market, 19 Oct 2003, *CJ128* (HKAS44340); Huidong County, Haiba, 2400m, 19 Oct 2003, *CJ126* (KUN-HKAS44317); Huidong County, Luozuo, 2200m, 21 Oct 2003, *CJ137* (KUN-HKAS44344); Miyi, 18 Nov. 1998, *Z. H. Mao & M. S. Yuan 4045* (KUN-HKAS33426); *Z. H. Mao 4046* (KUN-HKAS33438).Yunnan Prov.: Chuxiong city,Yongren County, 17 Nov. 2004, *CJ242* (HKAS47615); 2 Nov 2004 *CJ232* (KUN-HKAS47609); 25 Sept 2004, *CJ223* (KUN-HKAS45384); *CJ224* (KUN-HKAS45385); *CJ222* (KUN-HKAS45383); 14 Dec 2005, *CJ291* (KUN-HKAS49748); *CJ293* (KUN-HKAS49750); *CJ294* (KUN-HKAS49751); *CJ295* (KUN-HKAS49752); *CJ296* (HKAS49753); 18 Feb. 2000, *K. M. Su1* (KUN-HKAS34548); Xiangyun, under *Pinus yunnanensis*, Jan 2002, *D. S. Song 3* (KUN-HKAS39506); Lijiang city, Yulong Monutain, 2000-3000m, under *Pinus yunnanensis*, 3 Aug 1995, *M. Li & B. Li 1* (KUN-HKAS29357); 10 Nov 1990, *C. X. Pu2* (KUN-HKAS22914); *C. X. Pu 3* (KUN-HKAS22915). Lijiang city, Yongsheng county, 1925-2150m, 19 Oct 2004, *CJ228* (KUN-HKAS47612), *CJ227* (KUN-HKAS47608); *CJ215* (KUN-HKAS45386); *CJ216* (KUN-HKAS45389); *CJ217* (KUN-HKAS45387); *CJ218* (KUN-HKAS45390); *CJ219* (KUN-HKAS45380); Yongsheng county, Qina town, under *P. yunnanensis* and *Quercus acutissima*, *C. G. Xiang 9091* (KUN-HKAS22911); *C. G. Xiang 9092* (KUN-HKAS22912); 2460m, 21 Dec 2003, *CJ153* (KUN-HKAS44988); *CJ152* (KUN-HKAS44987); *CJ154* (KUN-HKAS 44989); 18 Sept 2004, *CJ214* (HKAS45381); *CJ213* (KUN-HKAS 45382); 3 Dec 2004, *CJ244* (KUN-HKAS47610); Gongshan, 20 Jan 2002, *D. S. Song 4* (KUN-HKAS39507); Gongshan county, Yonglaga, 1400m, under *Castanina* sp*.*, 27 Sept 2003, *CJ110* (KUN-HKAS44330); *CJ116* (KUN-HKAS44331); *CJ111* (KUN-HKAS44332); *CJ114* (KUN-HKAS44333). Gongshan county, Binzhongluo, 1400m, 26 Sept 2003, *CJ112* (KUN-HKAS44329); *CJ113* (KUN-HKAS44334); Gaoligong Montain, 25 Nov. 2002, *Z. L.Yang 3604* (KUN-HKAS41314); *Z. L.Yang 3605* (KUN-HKAS41315); *Z. L.Yang 3606* (KUN-HKAS41316); Kunming city, Haikou county, 2100m, 1 Dec 2002, *H. C. Wang 258* (KUN-HKAS42009); 2100m, 25 Nov 2002, *Z. L.Yang 3602* (KUN-HKAS41312); Zhaotong city, Huize county, under *P. yunnanensis*, 10 Sept 1992, *D. Pu 1* (KUN-HKAS25689), Kunming market, 25 Oct 2005, *CJ286* (KUN-HKAS49743); 8 Nov 2005, *CJ288* (HKAS49745); 23 Jan. 2002, *D. S. Song 2* (KUN-HKAS39503). Kunming city, Jindian, 13 Mar. 2003, *F. Q. Yu 1089* (KUN-HKAS44990); Western Montain, under *P. armandii*, 15 Jan 2004, *CJ156* (KUN-HKAS44999); 14 Nov 2003, *CJ149* (KUN-HKAS44339); Jan 2002, *D. S. Song 7* (KUN-HKAS39501); Jinning, 4 Nov 2004, *CJ237* (KUN-HKAS47606); *CJ236* (KUN-HKAS47607). Dongchuan, under *P. Yunnanensis*, 2500m, Nov 1996, *C. X. Pu 2* (KUN-HKAS30264); Dongchuan, under *P. densata*, 11 Mar 2003, *F. Q. Yu 1087* (KUN-HKAS42398); Chengong, 2000m, under *P. armandii*, 26 Nov. 1997, *M. Zang 12891*(KUN-HKAS30838a), *M. Zang 12891A* (KUN-HKAS30839); 1990m, under *P. yunnanensis*, 5 Sept 2000, *F. Q. Yu 377* (KUN-HKAS38933); Yimen, 2400m, 14 Dec 2005, *CJ289* (KUN-HKAS49746); 1 Sept 2000, *X. H. Wang 1179* (KUN-HKAS36863); Kunming market, 4 Apr 2004, *CJ157* (KUN-HKAS4937); 10 Nov. 2003, *CJ146* (KUN-HKAS44318); *CJ151* (KUN-HKAS44337); 8 Mar 2003, *F. Q. Yu 1086* (KUN-HKAS42397); *F. Q. Yu 1085* (KUN-HKAS42392); Kunming market, 22 Feb 2005, *CJ247* (KUN-HKAS48273); 20 Feb 2002, *Z. L. Yang 3220* (KUN-HKAS39515); *Z. L. Yang 3221* (KUN-HKAS39516); 9 Mar 2003, *anonymity 4* (KUN-HKAS42396); *anonymity 2* (KUN-HKAS42395); *anonymity 3* (KUN-HKAS42394); 23 Sept 2002, *F. Q. Yu1060* (KUN-HKAS41740); *H. D. Zheng 179* (KUN-HKAS42237); 4 Nov 2004, *CJ235* (KUN-HKAS47611); Baoshan city, 2475m, 8 Nov 2004, *CJ239* (KUN-HKAS47614). India – Uttar Pradesh, Mussoorie, 13 Jan 1892, *J. F. Duthie* K(M)39493 – **Holotype of *T. indicum****.*
