# Supplementary material for: Species Recognition and Cryptic Species in the Tuber indicum Complex
Source: PLoS One. 2011 Jan 28;6(1):e14625. doi: 10.1371/journal.pone.0014625 (PMC3030557; doi:10.1371/journal.pone.0014625)
Supplement: Table S1 — Tuber species used in the study and their geographic distribution. (0.22 MB DOC) [file pone.0014625.s002.doc]

**Table S1. *Tuber* species used in the study and their geographic distribution.**

| Taxa | Isolate | Locality | Host tree | Genbank# | | |
| --- | --- | --- | --- | --- | --- | --- |
|  |  |  |  | ITS | β-tubulin | LSU |
| ***Tuber aestivum**** | T3 | Huidong, Sichuan, China | *Pinus armandii* | GQ217542 | GU979150 |  |
| ***T. aestivum**** | T27 | Huidong, Sichuan, China | *P. armandii* |  | GU979151 | GU979101 |
| ***T. aestivum**** | T63 | Huidong, Sichuan, China | *P. armandii* | GU979038 | GU979152 |  |
| ***T. aestivum*** | E1 | Abruzzo, Italy | Unknown | AF516788 |  |  |
| ***T. aestivum*** | E17 | Abruzzo, Italy | Unknown | AY226042 |  |  |
| ***T. indicum**** | T82 | Chuxiong, Yunnan, China | *P. yunnanensis* | GU979060 | GU979175 | GU979128 |
| ***T. indicum**** | T36 | Yunnan, China | *P. yunnanensis* |  |  | GU979130 |
| ***T. indicum**** | T60 | Chuxiong, Yunnan, China | *P. yunnanensis* | GU979059 | GU979174 | GU979134 |
| ***T. indicum**** | T25 | Gongshan, Yunnan, China | *Castanea mollissima* | GU979058 | GU979172 | GU979114 |
| ***T. indicum**** | T52 | Gongshan, Yunnan, China | *C. mollissima* | GU979053 |  | GU979116 |
| ***T. indicum**** | CJ116 | Gongshan,Yunnan,China | *C. mollissima* |  |  | GU979131 |
| ***T. indicum**** | T51 | Gongshan,Yunnan,China | *C. mollissima* | GU979052 |  | GU979115 |
| ***T. sinense**** | T50 | Huidong, Sichuan, China | *P. armandii* | GU979063 |  |  |
| ***T. indicum**** | T15 | Kunming market, China | Unknown | GU979068 | GU979166 |  |
| ***T. indicum**** | T77 | Kunming Market, China | Unknown | GU979057 | GU979173 |  |
| ***T. indicum**** | T74 | Yongsheng, Yunnan, China | *P. yunnanensis* | GU979056 | GU979176 |  |
| ***T. indicum**** | T75 | Gongshan,Yunnan,China | *C. mollissima* | GU979054 | GU979178 |  |
| ***T. sinense**** | T68 | Huidong, Sichuan, China | *P. armandii* | GU979062 | GU979161 |  |
| ***T. indicum**** | T59 | Yongsheng, Yunnan, China | *P. yunnanensis* | GU979074 | GU979164 |  |
| ***T. sinense**** | T58 | Huidong, Sichuan, China | *P. armandii* | GU979072 | GU979163 |  |
| ***T. indicum**** | CJ223 | Chuxiong, Yunnan, China | *P. yunnanensis* |  |  | GU979132 |
| ***T. indicum**** | T10 | Paizhihua, Sichuan, China | Unknown | GU979050 |  | GU979112 |
| ***T. formosanum**** | T12 | Taiwan island, China | *Cyclobalanopsis glauca* | GU979048 | GU979158 |  |
| ***T. formosanum**** | T44 | Taiwan island, China | *C. glauca* | GU979049 | GU979159 | GU979126 |
| ***T. indicum**** | T46 | Yongsheng, Yunnan, China | *P. yunnanensis* | GU979055 |  | GU979125 |
| ***T. indicum**** | T66 | Paizhihua, Sichuan, China | Unknown | GU979051 | GU979177 | GU979113 |
| ***T. indicum**** | T113 | Huidong, Sichuan, China | *P. armandii* | GU979081 | GU979181 | GU979137 |
| ***T. indicum**** | T115 | Yongsheng, Yunnan, China | *P. yunnanensis* | GU979082 | GU979182 | GU979138 |
| ***T. indicum**** | T43 | Dongchuan, Yunnan, China | Unknown | GU979076 | GU979170 | GU979123 |
| ***T. indicum**** | T83 | Chuxiong, Yunan, China | *P. yunnanensis* |  | GU979168 | GU979129 |
| ***T. indicum**** | T39 | Huize,Yunnan, China | Unknown | GU979064 |  | GU979118 |
| ***T. indicum**** | T92 | Yimen, Yunnan, China | *P. yunnanensis* | GU979080 | GU979180 | GU979136 |
| ***T. sinense**** | T11 | Huidong, Sichuan, China | *P. armandii* | GU979061 | GU979160 | GU979117 |
| ***T. sinense**** | T48 | Huidong, Sichuan,China | *P. armandii* | GU979071 |  |  |
| ***T. indicum**** | T49 | Kunming, China | *P. yunnanensis* | GU979078 |  | GU979119 |
| ***T. indicum**** | T47 | Jinning, Yunnan, China | *P. yunnanensis* | GU979070 |  |  |
| ***T. indicum**** | CJ236 | Jinning, Yunnan, China | *P. yunnanensis* |  |  | Gu979133 |
| ***T. indicum**** | T81 | Yimen, Yunnan, China | *P. yunnanensis* | GU979067 | GU979167 | GU979127 |
| ***T. indicum**** | T45 | Yongsheng, China | *P. yunnanensis* | GU979075 |  | GU979121 |
| ***T. indicum**** | T90 | Chuxiong, Yunnan, China | *P. armandii* | Gu979079 | GU979179 | GU979135 |
| ***T. indicum**** | T16 | Haikou, Yunnan, China | Unknown | GU979069 | GU979169 | GU979122 |
| ***T. indicum**** | T26 | Kunming, Yunan, China | *P. yunnanensis* | GU979066 | GU979162 | GU979120 |
| ***T. indicum**** | T37 | Yongsheng, Yunnan, China | *P. yunnanensis* | GU979073 |  |  |
| ***T. indicum**** | T38 | Huize, Yunnan, China | Unknown | GU979077 | GU979171 | GU979124 |
| ***T. indicum**** | T72 | Kunming, Yunnan, China | Unknown | GU979065 | GU979165 |  |
| ***T. indicum*** | Ti20 | Unknown | Unknown | U89362 |  |  |
| ***T. sinense*** |  | Huidong, Sichuan, China | Unknown | DQ329376 |  |  |
| ***T. indicum*** | Ti38 | Unknown | Unknown | U89361 |  |  |
| ***T. indicum*** |  | Panzhihua, Sichuan, China | Unknown | DQ329366 |  |  |
| ***T.indicum*** |  | Panzhihua, Sichuan, China | Unknown | DQ375505 |  |  |
| ***T. indicum*** |  | Gongshan, Yunnan, China | Unknown | DQ375494 |  |  |
| ***T. indicum*** |  | Miyi, Sichuan, China | Unknown | DQ375499 |  |  |
| ***T. indicum*** |  | Miyi, Sichuan, China | Unknown | DQ375501 |  |  |
| ***T. indicum*** |  | Unknown | Unknown | AF106882 |  |  |
| ***T.indicum*** | HKAS  39516 | Kunming, Yunnan, China | Unknown | AY514308 |  |  |
| ***T.indicum*** |  | Huili, Sichuan, China | Unknown | DQ375498 |  |  |
| ***T.indicum*** |  | Yunnan market, China | Unknown | DQ375518 |  |  |
| ***T.indicum*** |  | Yunnan market, China | Unknown | DQ375510 |  |  |
| ***T.indicum*** |  | Yunnan market, China | Unknown | DQ375524 |  |  |
| ***T.sinense*** |  | Huidong, Sichuan, China | Unknown | DQ375527 |  |  |
| ***T. himalayense*** | HKAS  25689 | Huize, Yunnan, China | Unknown | AY773356 |  |  |
| ***T. indicum*** | Ti17 | Huili, Sichuan, China | Unknown | AF132502 |  |  |
| ***T. melanosporum*** |  | Unknown | Unknown |  |  | AF435821 |
| ***T. melanosporum* *** | T99 | Italy | Unknown | GU979083 | GU979183 | GU979139 |
| ***T. melanosporum*** | A59 | France | Unknown | AF106878 |  |  |
| ***T.melanosporum*** |  | Aquila, Italy | Unknown | U89359 |  |  |
| ***T. melanosporum*** |  | Tarn, Albigeois, France | Unknown | AJ583651 |  |  |
| ***T. melanosporum*** | Tm13 | Vaucluse, France | Unknown | AF132501 |  |  |
| ***T. melanosporum*** | MEL  142 | Unknown | Unknown |  | AY170365 |  |
| ***T. pseudohimalayense**** | T41  Isotype | Import from China | Unknown |  |  | GU979110 |
| ***T. pseudoexcavatum**** | T14 | Huidong, Sichuan, China | Unknown | GU979039 | GU979153 | GU979102 |
| ***T. pseudoexcavatum**** | T29 | Huidong, Sichuan, China | Unknown | GU979040 | GU979154 | GU979103 |
| ***T. pseudoexcavatum**** | T80 | Yimen, Yunnan, China | *P. yunnanensis* | GU979046 | GU979157 | GU979107 |
| ***T. pseudoexcavatum**** | T33 | Huidong, Sichuan, China | *P. armandii* |  |  | GU979108 |
| ***T. pseudoexcavatum**** | T114 | Huidong, Sichuan, China | *P. armandii* | GU979042 |  | GU979109 |
| ***T. pseudoexcavatum**** | T13 | Huidong, Sichuan, China | *P. armandii* | GU979041 |  | GU979104 |
| ***T. pseudoexcavatum**** | T31 | Baoshan, Yunnan, China | *P. armandii* | GU979045 | GU979156 | GU979106 |
| ***T. pseudoexcavatum**** | T32 | Kunming, Yunnan, China | Unkown | GU979044 |  |  |
| ***T. pseudoexcavatum**** | T18 | Kunming, Yunnan, China | Unkown | GU979043 | GU979155 | GU979105 |
| ***T. pseudoexcavatum*** | HKAS  39504 | Chuxiong, Yunnan, China | Unknown | AY514310 |  |  |
| ***T. brumale*** |  | Marche, Italy | Unkown | AF106880 |  |  |
| ***T. brumale*** |  | Unknown | Unkown | AF132504 |  |  |
| ***T. borchii*** |  | Unknown | Unkown | AF250291 |  |  |
| ***T. borchii*** |  | Unknown | Unkown | AF106890 |  |  |
| ***T. borchii*** |  | Modena, Italy | Unkown | AY940651 |  |  |
| ***T. excavatum**** | T106 | Huidong, Sichuan, China | Unkown | GQ217540 | GU979148 |  |
| ***T. excavatum*** |  | Unkown | Unkown |  |  | DQ191677 |

*Sequences obtained in our lab.
